# Effects of Macronutrient Distribution on Weight and Related Cardiometabolic Profile in Healthy Non-Obese Chinese: A 6-month, Randomized Controlled-Feeding Trial

**DOI:** 10.1016/j.ebiom.2017.06.017

**Published:** 2017-06-20

**Authors:** Yi Wan, Fenglei Wang, Jihong Yuan, Jie Li, Dandan Jiang, Jingjing Zhang, Tao Huang, Jusheng Zheng, Jim Mann, Duo Li

**Affiliations:** a Department of Food Science and Nutrition, Zhejiang University, Hangzhou, China; b No. 1 Department of Nutrition, Chinese People's Liberation Army General Hospital, Beijing, China; c Saw Swee Hock School of Public Health, National University of Singapore, Singapore; d Department of Human Nutrition and Medicine, University of Otago, Dunedin, New Zealand; e Institute of Nutrition and Health, Qingdao University, Qingdao, China

**Keywords:** Diet, Macronutrient composition, High fat, Low carbohydrate, Body weight, Cardiometabolic profile

## Abstract

**Background:**

It has been suggested that the increase in carbohydrate at the expense of fat has contributed to the obesity epidemic in North America and some European countries. However, obesity rates in China have increased rapidly in parallel with a transition from the traditional low fat, high carbohydrate diet to a diet relatively high in fat and reduced in carbohydrate. Therefore, the current study aimed to determine whether the traditional Chinese diet was likely to be more effective than a diet with higher fat and lower carbohydrate — which is consumed in most Western societies, at weight control among a non-obese healthy population in China.

**Methods:**

The 6-month, two-center, three-arm, randomized, parallel-group, controlled-feeding trial was conducted at People's Liberation Army General Hospital in north China and Zhejiang University in south China. We recruited healthy young adults (aged 18–35 years, body mass index < 28) who lived in the university campus or the hospital dormitory during the whole study intervention period. They were required to eat only the foods provided, and to avoid excessive or unusual strenuous exercise during the trial. Participants were simultaneously enrolled and randomized using a computer-generated number (stratified by clinic center, age, sex, and body mass index) by data manager to one of the three isocaloric diets (1:1:1): a lower fat, higher carbohydrate diet (fat 20%, carbohydrate 66% energy); a moderate fat, moderate carbohydrate diet (fat 30%, carbohydrate 56% energy); a higher fat, lower carbohydrate diet (fat 40%, carbohydrate 46% energy). Protein provided 14% energy in all diets. We provided all food and beverages throughout the 6-month intervention. Laboratory personnel were masked to treatment allocation. Body weight was the primary outcome and measured each month. Data were primarily analyzed according to an intention-to-treat approach, supplemented with per-protocol analysis. The study was approved by the Ethics Committee at Zhejiang University. Each participant provided written informed consent. The study was registered at Clinicaltrials.gov, number NCT02355795.

**Findings:**

Between April 30, 2016, and October 30, 2016, 307 participants were randomly assigned to the lower fat diet (n = 101), the moderate fat diet (n = 105) and the higher fat diet (n = 101), and 245 (79.8%) participants completed the study. Reduction in body weight was significantly greater in the lower fat, higher carbohydrate group throughout the intervention (P < 0.001 for the interaction between diet group and time) than in the two other groups. Weight change at 6 months was − 1.6 kg (95% CI − 1.8 to − 1.4) in the lower fat, higher carbohydrate group; − 1.1 kg (95% CI − 1.3 to − 0.9) in the moderate fat, moderate carbohydrate group, and − 0.9 kg (95% CI − 1.1 to − 0.6) in the higher fat, lower carbohydrate group. Reduction in waist circumference, total cholesterol, low-density lipoprotein cholesterol and non-high-density lipoprotein cholesterol on the lower fat, higher carbohydrate group were greater than those observed on the other two diet groups.

**Interpretation:**

A lower fat, relatively higher carbohydrate diet, similar in macronutrient composition to that traditionally eaten in China appears to be less likely to promote excessive weight gain and be associated with a lower cardiometabolic risk profile than a diet more typical of that eaten in Western countries in healthy non-obese Chinese. Findings from studies in European and North American populations suggesting possible benefits of carbohydrate restriction may not apply to people of other ethnicities.

## Highlights

• A LF-HC diet was associated with a greater weight loss and great reduction in waist circumference than a higher fat, lower carbohydrate diet.
• Cardiometabolic risk profiles were more favorable on the LF-HC diet than on a higher fat, lower carbohydrate diet.
• Measures of glycemia were unchanged throughout the trial, regardless if the macronutrient composition.

This is a controlled-feeding trial to evaluate the effects of altering dietary fat and carbohydrate intake on weight change among non-obese subjects in a country experiencing nutritional transition. All the foods and most beverages were provided in order to maximize dietary targets and reduce other dietary or lifestyle related confounding factors during the intervention period. Our findings in conjunction with all other available evidence suggest that the Chinese population should be discouraged from following the continuing trend towards increasing dietary fat intake at the expense of carbohydrate.

## 1 Introduction

In the United States and many western countries, the prevalence of obesity and type 2 diabetes (T2D) has increased dramatically in parallel with a reduction in total fat and an increase in carbohydrate intake in the past several decades (Austin et al., 2011). This dietary change is considered by some to, at least in part, have contributed to the substantial increase in rates of obesity and T2D (Mozaffarian et al., 2011, Livesey et al., 2013, Te Morenga et al., 2013). Findings from several relatively short-term clinical trials and observational studies suggested that diets characterized by moderate restriction of carbohydrate, but relatively high in unsaturated fat improved blood lipid profile and lowered coronary heart disease (CHD) risk among individuals with high cardiometabolic risk (Jenkins et al., 2009, Halton et al., 2006). Some investigators have suggested that carbohydrate restriction rather than fat reduction should be recommended as the most appropriate nutritional approach to achieve overall reduction in obesity and cardiometabolic risk (Hu, 2010).

However, a very different situation prevails in China where over a similar period consequences of overnutrition have replaced those of malnutrition (Du et al., 2014, Zhai et al., 2014). Whereas some thirty years ago, rates of overweight and obesity were extremely low compared with the rest of the world, now nearly half the Chinese population is overweight and the majority are at high risk of cardiometabolic disease (Adair et al., 2014). Unlike the situation in the United States, from 1982 to 2011, energy intake from fat doubled from 18% to 32% (37% in Chinese megacities) and daily edible oil nearly tripled from 18 to 49 g (Jenkins et al., 2009, Ministry of Health: Bureau of Disease Control and Prevention, 2011). Correspondingly, total daily energy from carbohydrate decreased significantly from 72% to 54% (47% in Chinese megacities) (Jenkins et al., 2009). Ecologic studies suggested that increased fat intake and corresponding reduction in carbohydrate might be an important contributing factor to increases in obesity and cardiometabolic disease (Campbell, 2004, Popkin et al., 1995). On the other hand, recent prospective cohort studies indicated that intake of carbohydrate, particularly white rice and highly processed wheat products are associated with increased CHD and T2D risk in Chinese adults (Yu et al., 2013, Villegas et al., 2007).

Given the different trends in fat and carbohydrate consumption in the Chinese population and populations in North America and Europe, despite a common trend towards increased rates of obesity and its comorbidities, we decided to conduct a randomized, controlled trial among our population to determine whether the traditional lower fat, higher carbohydrate diet or the western higher fat, lower carbohydrate diet is more effective at weight control and related cardiometabolic profile in modern Chinese.

## 2 Materials and Method

### 2.1 Study Design

This study was an observer-blinded, parallel, three-arm, randomized controlled-feeding trial conducted at People's Liberation Army General Hospital in north China and Zhejiang University in south China and was approved by the Ethics Committee at Zhejiang University. The study protocol is published (Wan et al., 2017). The trial is registered at ClinicalTrials.gov, number NCT02355795.

### 2.2 Participants, Randomization and Masking

Recruitment began in January 25, 2015. Trial participants were healthy young adults and were recruited primarily using fliers and internet advertisements. Eligibility for the trial was determined from 3 screening visits and a 7-day run-in period (Table S1). Key inclusion criteria included overall good health, residence on the university campus or hospital dormitory, age range between 18 and 35 years and body mass index (BMI) < 28 according to the Chinese obesity criteria. Key exclusion criteria included blood pressure ≥ 140 mmHg systolic or 90 mmHg diastolic, total, LDL cholesterol ≥ 6.19 mmol/L and 4.12 mmol/L respectively, fasting triglyceride ≥ 2.25 mmol/L, fasting glucose ≥ 6.11 mmol/L, change in body weight exceeding 10% during the previous year and pre-existing chronic diseases. After the 3 screening visits, all volunteers participated in breakfast acceptability tests. They were provided with sample breakfast foods (lower fat bread, moderate fat cookies, higher fat cookies) identical to those which would be provided throughout the trial. After that, participants entered into a 7-day run-in period during which they were fed the moderate fat, moderate carbohydrate (MF-MC) diet.

Upon successful completion of run-in period, eligible participants were randomized (1:1:1) to either a lower fat, higher carbohydrate (LF-HC) diet, a MF-MC diet, or a higher fat, lower carbohydrate (HF-LC) diet, stratified by clinic center, age, sex, and BMI using a computer-generated random number list. Clinical staff and laboratory personnel who did the measurements were masked to group allocation. Meal providers were aware of participant diet assignment, but they were not involved in the rest of the trial, including later measurement and results analyses. Due to the obvious difference in the breakfast provided, blinding participants was not feasible, though they were not informed of the allocated treatment.

### 2.3 Procedures

The three diets were isocaloric, the primary distinguishing feature being their fat and carbohydrate content (Table 1). By replacing a proportion of energy derived from carbohydrates (white rice and wheat flour, the most consumed carbohydrate sources in China contributing to 70% and 17% total carbohydrate respectively) with fats (soybean oil, the most consumed edible oil in China rich in unsaturated fatty acids), we achieved the required distribution of fats and carbohydrates in the three diet groups, which represented macronutrient transition in the past 30 years in China. The LF-HC diet (fat 20%, carbohydrate 66% energy) corresponds to the macronutrient distribution 30 years ago during which obesity was rare in China; the MF-MC diet (fat 30%, carbohydrate 56%) is based on the current macronutrient intake in China and also the upper limit of fat intake recommended by the Chinese Nutrition Society and (Chinese Dietary Reference Intakes, 2014); and the HF-LC diet (fat 40%, carbohydrate 46%) approximates the current consumption of Chinese residents of some megacities. Protein provided 14% energy in all diets. A sample, one-day set of meals is shown in Table 2.

**Table 1:** Nutrient targets and menu analysis of the 3 study diets.

Nutrients	LF-HC diet		MF-MC diet		HF-LC diet	
	Targets	Menu analysis^a^	Targets	Menu analysis^a^	Targets	Menu analysis^a^
Total energy (male), kcal^b^	2100	2094	2100	2096	2100	2103
Total energy (female), kcal^b^	1700	1697	1700	1698	1700	1704
Carbohydrate, %	66	66	56	55	46	46
Fat, %	20	20 (18)	30	31 (28)	40	40 (38)
Protein, %	14	14 (12)	14	14 (11)	14	14 (12)
Dietary fiber, g	14	14	14	13	14	14
Cholesterol, mg	300	289	300	289	300	289

LF-HC = lower fat, higher carbohydrate. MF-MC = moderate fat, moderate carbohydrate. HF-LC = higher fat, lower carbohydrate.

a Values were calculated using Nutrition System of Traditional Chinese Medicine Combining with Western Medicine, version 11.0 (Medical College, Qingdao University, Shandong, China). The nutrition system includes food composition data, permitting calculation of nutrient intake from the menus. Values in the parentheses are the results of chemical analyses of the menus prepared during the intervention period.

b Targets energy intake was determined from the 3-day dietary record at baseline.

**Table 2:** One-day sample menu for the female.^a^

Meal^b^	LF-HC diet			MF-MC diet			HF-LC diet		
	Food item		g	Food item		g	Food item		g
Breakfast^c^	Soymilk		250	Soymilk		250	Soymilk		250
	Low-fat bread:	Flour	58	Moderate-fat cookies:	flour	50	High-fat cookies:	flour	48
		Sugar	16		Soybean oil	20		Soybean oil	38
		Egg yolk	7		Sugar	4		Sugar	4
					Egg white	30		Egg white	65
					Egg yolk	7		Egg yolk	7
								Bran	2
Lunch	Rice		120	Rice		115	Rice		80
	Dish A:	Pork chop	44	Dish A:	Pig chop	44	Dish A:	Pig chop	44
		Shallot	5		Shallot	5		Shallot	5
	Dish B:	Tofu	40	Dish B:	tofu	40	Dish B:	Tofu	40
		Chub	48		Chub	48		Chub	48
	Dish C:	White gourd	60	Dish C:	White gourd	60	Dish C:	White gourd	60
		Cucumber	200		Cucumber	200		Cucumber	200
	Soybean oil		8	Soybean oil		8	Soybean oil		8
	Salt		2	Salt		2	Salt		2
Dinner	Rice		120	Rice		90	Rice		75
	Dish A:	Chicken wing	88	Dish A:	Chicken wing	88	Dish A:	Chicken wing	88
		Ginger	2		Ginger	2		Ginger	2
	Dish B:	tomato	36	Dish B:	tomato	36	Dish B:	tomato	36
		egg	18		egg	18		Egg	18
	Dish C:	Chinese chives	100	Dish C:	Chinese chives	100	Dish C:	Chinese chives	100
	Soybean oil		8	Soybean oil		8	Soybean oil		8
	Salt		2	Salt		2	Salt		2

LF-HC = lower fat, higher carbohydrate. MF-MC = moderate fat, moderate carbohydrate. HF-LC = higher fat, lower carbohydrate.

a For the male, the amount of all food items, except soybean milk, were multiplied by 1.2 based on their baselined energy intake.

b Traditional Chinese cuisines include a staple (rice or noodles or steamed bread) and stir-fried dishes (mixed food with edible oil). A majority of Chinese people tend to leave a little edible oil unconsumed which is unable to be quantitatively controlled. In order to alter the fat content of the diet without participants having to make major eating behavior, we did not distribute the additional fat evenly over the meals, but only delivered at breakfast to be consumed as low-fat bread or higher fat cookies. For example, at the energy level for the females, the LF-HC group had low-fat bread with 6.5 g of fat, while the MF-MC group replaced the low-fat bread with cookies containing 26.2 g of fat and the HF-LC group did the same but their cookies contained 44.2 g of fat. Otherwise, the menus for the 3 groups were almost identical, apart from the MF-MC and HF-LC groups eating less rice at lunch and dinner than the low-fat group. To enhance the public health relevance of the trial, the test diets were constructed with naturally occurring foods and did not include supplements or unfamiliar foods. The meals in the menus were prepared in duplicate, composited, and frozen for chemical analysis of fat and protein. For production consistency and quality control, each site purchased the same brand of each particular food item, and soybean oil was the only edible oil used in food preparation. Standardized recipes and cooking procedures were meticulously followed under sanitary conditions. As most Chinese dishes were mixed food with varied garnishes, each item was prepared in batch quantities, individually portioned, and weighed to within 0.1 g for portions < 10 g or within 0.5 g for those ≥ 10 g using electronic scales.

c In order to balance the intake of dietary cholesterol, breakfast in LF-HC received an egg yolk (part of the egg which is high in cholesterol), whereas the other groups received a whole egg for breakfast. In order to balance the intake of dietary fiber, breakfast in HF-LC received bran whereas the other diets did not.

Throughout the 6-month intervention, participants were provided with all of their food. Six 7-day menus cycling (each for one month) at two energy levels (one for female and one for male, according to average baseline energy intake) were developed for each diet. Participants ate at least one meal (lunch or dinner, packaged) on-site, where they were monitored by staff, while breakfast was packaged for consumption off-site. Participants were advised to maintain the same fruit consumption and physical activity level as before the trial. During the controlled feeding, they were asked to complete a daily diary in which they recorded whether they had eaten all study foods and detailed non-study foods eaten. At the end of each month, participants reported an overall satisfaction with the diet program using a 10-cm visual analog scale with appropriate verbal anchors.

### 2.4 Outcomes

All data were collected at baseline and monthly thereafter. The primary outcome was weight change from baseline to 6 months. Secondary outcomes were the changes of waist circumference, blood pressure, lipid profile (total, LDL, and HDL cholesterol, triglycerides, apolipoprotein A1, and apolipoprotein B), glycemic traits (glucose, glycated serum protein, and insulin), adiponectin, and leptin from baseline to 6 months.

Dietary intake data at baseline were collected by a 3-day dietary record (2 weekdays and 1 weekend day) using Nutrition System of Traditional Chinese Medicine Combining with Western Medicine, version 11.0 (Medical College, Qingdao University, Shandong, China). The nutrition system includes food composition data, permitting calculation of nutrient intake from reported food intake. Physical activity at each time point was assessed by the Global Physical Activity Questionnaire developed by WHO (GPAQ Analysis Guide, 2014).

### 2.5 Statistical Analyses

Main analyses followed the intention-to-treat approach. The primary outcome, body weight, was analyzed by mixed-model analysis of variance without imputing missing values (UCLA Statistical Consulting, 2016). Within-participant correlation was accounted for by a random effect (repeated measures with compound-symmetric covariance). Age, sex and study center were included as covariates. Mean changes derived from mixed models at each time point were used for graphical presentation. The group × time interaction term provides a test of the hypothesis that body weight among groups do not differ across the study period. A parallel approach was used for secondary outcomes including waist circumference, blood pressure, lipid profile (total, LDL, and HDL cholesterol, total/HDL cholesterol, non-HDL cholesterol, triglycerides, apolipoprotein A1, and apolipoprotein B), glycemic traits (glucose, glycated serum protein, and insulin), adiponectin, and leptin. Analysis for body weight was supplemented with a per-protocol method, while analyses of these secondary outcomes were only performed based on intention-to-treat principles. For exploratory purposes, ancillary analyses were conducted after adjusting for change in weight in mixed-model. Each of the statistically significant outcomes among groups across the intervention period remained statistically significant after including weight change in the model. For simplicity, only results without adjustment are presented. Also for exploratory purpose, multiple imputation analysis was performed for all outcomes. There was no qualitative difference of multiple imputation compared with values from mixed-model and complete case analysis. Full details are given in Fig. S1.

The power calculation was based on a between-group difference in weight change of 1.8 kg (approximately 3% for a 60-kg individual) and a 6.3-kg SD of weight change, based on a previous study and considered to be clinically meaningful (Gardner et al., 2007). The estimated 240 subjects provided a power of 90% to detect this difference in weight change at a two-tailed significance level of 0.05 and the sample size was then increased to 300 to take into account a possible loss-to-follow-up rate of 20%. All statistical analyses were carried out using SAS version 9.3 (SAS Institute Inc) for Windows.

## 3 Results

**Fig. 1:**
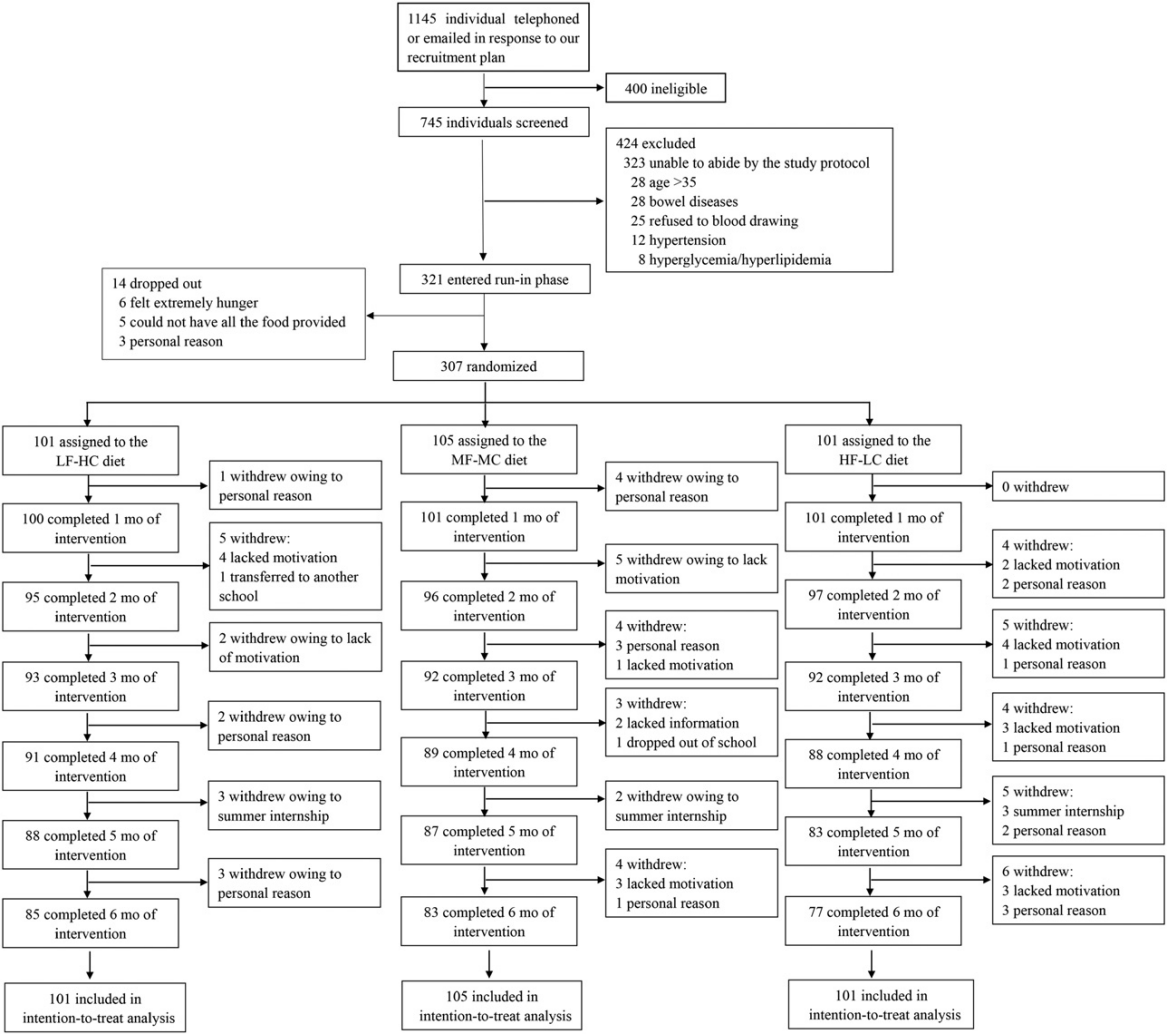
Trial profile. LF-HC = lower fat, higher carbohydrate. MF-MC = moderate fat, moderate carbohydrate. HF-LC = higher fat, lower carbohydrate.

Between 25 January 2015 and 30 October 2015, 1145 individuals initially telephoned or emailed in response to our recruitment, and 745 (65.1%) entered into the 3 screening visits for eligibility according to the inclusion and exclusion criteria. Of these individuals, 424 (56.9%) were excluded with recorded reasons. The remaining 321 entered into the run-in phrase. Upon successful completion of the run-in, 307 participants were randomized (n = 101 in the LF-HC group, n = 105 in the MF-MC group and n = 101 in the HF-LC group) and 245 (79.8%) completed the study (Fig. 1). Retention rates at 6 months were 84.2%, 79.0%, and 76.2% for the LF-HC, MF-MC, and HF-LC diet group, respectively. Baseline characteristics of participants are shown in Table 3. All 307 participants were included in the intention-to-treat analysis.

**Table 3:** Baseline characteristics of the intention-to-treat population.

	LF-HC diet (n = 101)	MF-MC diet (n = 105)	HF-LC diet (n = 101)
Age (years)	23.4 (3.6)	23.2 (3.9)	23.7 (4.3)
Female	51 (50%)	55 (52%)	49 (48%)
Northern area	49 (49%)	56 (53%)	47 (46%)
Weight (kg)	60.4 (10.4)	59.6 (10·1)	62.2 (10.5)
BMI (kg/m^2^)	21.7 (2.5)	21.8 (2.6)	21.9 (2.5)
Blood pressure (mm Hg)			
Systolic	117.0 (10.7)	115.5 (9.3)	116.0 (9.5)
Diastolic	70.9 (7.1)	69.5 (6.6)	71.1 (7.0)
Waist circumference (cm)	76.4 (10.0)	76.8 (9.4)	77.8 (9.9)
Blood biomarkers			
Total cholesterol (mmol/L)	4.2 (0.8)	4.1 (0.7)	4.1 (0.6)
LDL cholesterol (mmol/L)	2.4 (0.6)	2.4 (0.6)	2.4 (0.5)
HDL cholesterol (mmol/L)	1.5 (0.3)	1.5 (0.3)	1.5 (0.3)
Total/HDL cholesterol	2.8 (0.6)	2.9 (0.7)	2.9 (0.6)
Non-HDL cholesterol (mmol/L)	2.7 (0.7)	2.6 (0.7)	2.7 (0.6)
Triglycerides (mmol/L)	0.8 (0.4)	0.8 (0.4)	0.8 (0.4)
Apolipoprotein A1 (mmol/L)	1.6 (0.2)	1.6 (0.2)	1.6 (0.2)
Apolipoprotein B (mmol/L)	0.7 (0.2)	0.7 (0.2)	0.7 (0.1)
Glucose (mmol/L)	4.2 (0.5)	4.1 (0.5)	4.1 (0.6)
Glycated serum protein (μmol/L)	258.0 (17.2)	257.8 (16.2)	256.6 (17.7)
Insulin (pmol/mL)	71.0 (23.0)	70.3 (25.1)	70.3 (24.4)
Adiponectin (mg/L)	11.6 (4.0)	11.7 (4.4)	12.0 (4.1)
Leptin (μg/L)	5.0 (3.3)	4.9 (3.5)	5.0 (3.5)
Dietary intakes			
Energy (male, kcal/day)	2099.0 (128.8)	2097.9 (123.8)	2082.6 (107.1)
Energy (female, kcal/day)	1688.3 (97.7)	1705.5 (89.3)	1696.8 (96.6)
Total fat (% of TE)	30.3 (5.0)	31.0 (4.2)	31.0 (4.4)
Carbohydrate (% of TE)	55.6 (5.0)	55.1 (4.1)	54.8 (4.4)
Protein (% of TE)	14.0 (1.9)	14.0 (1.9)	14.2 (1.9)
Cholesterol (mg/day)	389.8 (354.0)	370.3 (303.5)	363.5 (314.8)
Fiber (g/day)	13.5 (5.6)	13.1 (5.5)	13.5 (5.5)
Physical activity (kcal/kg/h)	3.4 (1.7)	3.3 (2.0)	3.0 (1.5)

Data are mean (SD) or n (%). LF-HC = lower fat, higher carbohydrate. MF-MC = moderate fat, moderate carbohydrate. HF-LC = higher fat, lower carbohydrate. BMI = body mass index. LDL = low-density lipoprotein. HDL = high-density lipoprotein. TE = total energy.

Participants reported a high level of satisfaction with diets throughout the study with satisfaction scores ranging between 8 and 8.5 on a 10-cm visual analog scale (a score of 10 suggesting extremely satisfied) and no difference among groups. They also reported a high level of adherence to meals provided (93–98%) regardless of whether or not they were consumed under supervision (Fig. S2). Total energy (TE) intake (including study and non-study food eaten), percentage TE from macronutrients and amounts of dietary fiber and cholesterol calculated from 3-day diet records, and physical activity level are shown in Fig. S3.

**Fig. 2:**
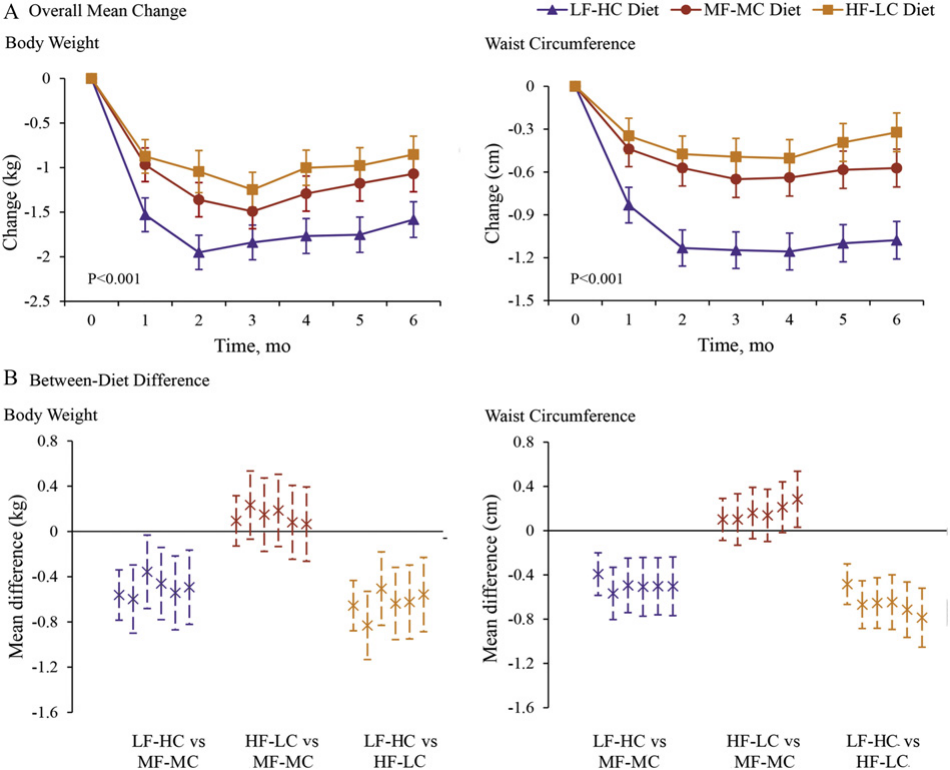
Mean changes and diet contrast in body weight and waist circumferences. Panel A shows mean changes in body weight and waist circumference. Error bars indicate 95% confidence intervals. Data are based on mixed-model analysis of variance. The P value at the lower left indicates the test of whether the change between baseline and intervention period (mean of each month) differed significantly between participants assigned to three diet groups. The P values for the comparison between the LF-HC group and the MF-MC group are < 0.001 for body weight and < 0.001 for waist circumference. The P values for the comparison between the HF-LC group and the MF-MC group are 0.44 for body weight and 0.21 for waist circumference. The P values for the comparison between the LF-HC group and the HF-LC group are < 0.001 for body weight and < 0.001 for waist circumference. Panel B shows the between-diet differences in body weight and waist circumference at 1 to 6 month. Error bars indicate 95% confidence intervals. LF-HC = lower fat, higher carbohydrate. MF-MC = moderate fat, moderate carbohydrate. HF-LC = higher fat, lower carbohydrate.

All groups lost weight and reductions were greater in the LF-HC group than in the MF-MC and HF-LC groups (P < 0.001 for the interaction between diet group and time) (Fig. 2). In the intention-to-treat analysis, 6-month weight changes among the 307 participants were − 1.6 kg (95% confidence interval [CI] − 1.8 to − 1.4) for the LF-HC group, − 1.1 kg (95% CI − 1.3 to − 0.9) for the MF-MC group, and − 0.9 kg (95% CI − 1.1 to − 0.6) for the HF-LC group. Among the 245 participants who completed the intervention, overall weight changes were − 1.6 kg (95% CI − 1.7 to − 1.4) for the LF-HC group, − 1.1 kg (95% CI − 1.3 to − 0.8) for the MF-MC group, and − 1.0 kg (95% CI − 1.3 to − 0.7) for the HF-LC group (Table S2). Similarly, all groups showed significant decreases in waist circumference, but the reductions were greater in the LF-HC group (P < 0.0001 for the interaction between diet group and time) than in the MF-MC group and HF-LC group (Fig. 2).

**Fig. 3:**
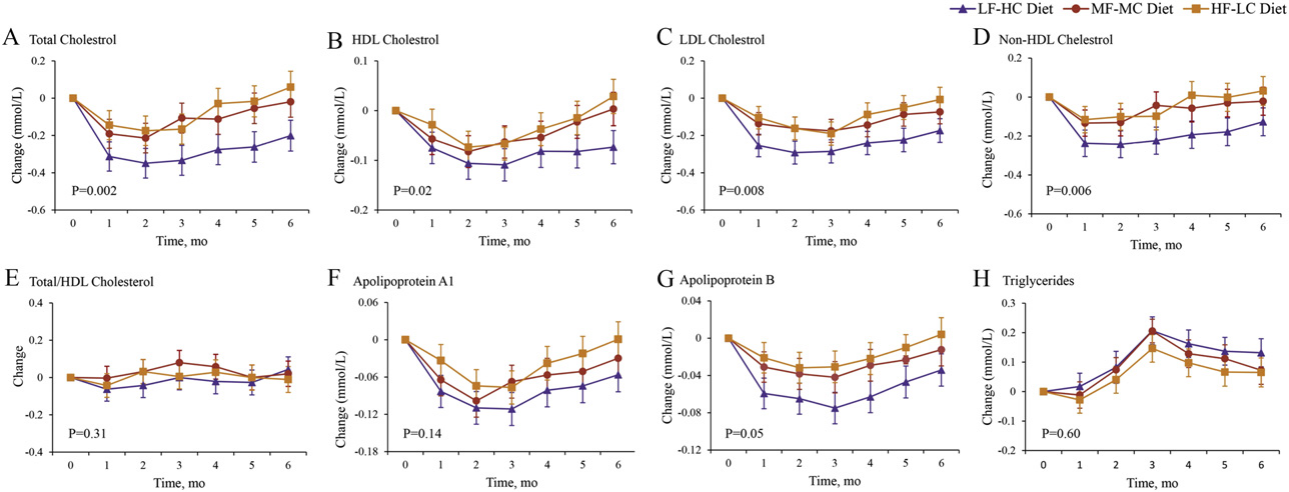
Mean changes in lipid profile throughout the 6-month intervention. Panel A shows the results for total cholesterol, Panel B for HDL cholesterol, Panel C for LDL cholesterol, Panel D for non-HDL cholesterol, Panel E for total/HDL cholesterol, Panel F for apolipoprotein A1, Panel G for apolipoprotein B, Panel H for triglyceride. Data are based on mixed-model analysis of variance. The P value at the lower left indicates the test of whether the change between baseline and intervention period (mean of each month) differed significantly between participants assigned to three diet groups. The P values for the comparison between the LF-HC group and the MF-MC group are 0.004 for total cholesterol, 0.03 for HDL cholesterol, 0.05 for LDL cholesterol, and 0.03 for non-HDL cholesterol. The P values for the comparison between the HF-LC group and the MF-MC group are 0.19 for the total cholesterol, 0.77 for HDL cholesterol, 0.48 for LDL cholesterol, and 0.27 for non-HDL cholesterol. The P values for the comparison between the LF-HC group and the HF-HC group are < 0.001 for total cholesterol, 0.004 for HDL cholesterol, 0.001 for LDL cholesterol, and 0.003 for non-HDL cholesterol. LF-HC = lower fat, higher carbohydrate. MF-MC = moderate fat, moderate carbohydrate. HF-LC = higher fat, lower carbohydrate.

Total, LDL, and non-HDL cholesterol decreased throughout the intervention, with the greatest decrease in the LF-HC group compared with the MF-MC and HF-LC groups (P values for the interaction between diet group and time were < 0.001, 0.009, and 0.006 for total, LDL, and non-HDL cholesterol, respectively, Fig. 3). While HDL cholesterol decreased to a greater extent in the LF-HC group compared with other two groups, the change in ratio of total cholesterol to HDL cholesterol among all groups did not differ significantly (Fig. 3). Other lipid profile including triglycerides, apolipoprotein A1, and apolipoprotein B also did not differ significantly among all groups throughout the intervention (Fig. 3).

All groups had small but significant decreases in blood pressure and glycated serum protein, but differences between groups were not significant. Fasting glucose, insulin, adiponectin and leptin did not change significantly during the course of the study and there were no significant differences among groups. Details are shown in Table S3.

## 4 Discussion

While all three dietary interventions were associated with decreases in weight, waist circumference, level of total, LDL, and non-HDL cholesterol, reductions in the LF-HC group were greater than those observed in the MF-MC and the HF-LC groups. HDL cholesterol was also reduced but there were no differences among groups with regard to total to HDL cholesterol ratio. Insulin and glucose levels remained unchanged throughout the trial. Thus, the traditional LF-HC diet consumed over a prolonged period confers benefit in terms of body weight and associated cardiometabolic risk factors when compared with diets with a macronutrient distribution more like those consumed in Western countries in healthy non-obese Chinese. Findings from studies in European and North American populations suggesting possible benefits of carbohydrate restriction may not apply to people of other ethnicities.

The finding with regard to body weight is consistent with that of a recent systematic review and meta-analysis of 33 randomized controlled trials and 10 cohort studies which compared, in individuals not attempting to lose weight, the effects of diets in which total fat provided < 30% TE with those providing higher intakes of total fat (Hooper et al., 2015). That analysis showed a modest but meaningful reduction of body weight (1.6 kg [95% CI − 2.0 to − 1.2]) on the lower fat diets, comparable with that observed in the present study. The systematic review was unable to identify any comparable studies addressing this issue in developing or transitional countries, a gap now filled by the present study. Trials involving obese people attempting to lose weight were excluded from the Hooper et al. meta-analysis as it was undertaken in order to generate a recommendation regarding the population impact of total fat intake in the development of obesity. Studies which were specifically designed to achieve weight loss in selected obese people would have limited application to non-obese populations or those in developing countries or countries experiencing nutritional transition.

In the present study, the finding that all three diet groups lost body weight was unexpected. This may have been due to the underestimation of total energy intake at baseline which provided the information on which food was provided for individual participants. Total energy provided in the experimental diet failing to meet the energy requirements might lead to the weight loss in all groups. Even prospective methods like the dietary records we have used may have limitations and in particular tend to underestimate intake of total energy (Macdiarmid and Blundell, 1998). However, the fact that the LF-HC group reduced body weight and improved blood lipid profile to a greater extent than the other two diet groups for whom energy intake was similarly calculated suggests potentially important clinical and public health implications. Not only is the traditional diet likely to be more effective in promoting weight loss in the overweight and obese but the traditional dietary pattern may also have greater potential than higher fat diets to reduce the risk of inappropriate weight gain in adult life. Few studies have investigated the underlying mechanisms by which macronutrient composition might influence energy balance independently on calculated energy intake. The high food efficiency of dietary fat provides a possible explanation. Diet-induced thermogenesis produces a loss of energy for the body which is 2–3% for fats and 6–8% for carbohydrates. Thus the efficiency of nutrient utilization is modestly higher for fats (97–98%) than for carbohydrates (92–94%) (Hariri and Thibault, 2010). Furthermore, dietary modification has the potential to alter the balance of the gut microbiota and therefore influence their capacity to harvest energy from the diet, which may have an impact on host energy balance (Turnbaugh et al., 2006). Further studies are needed to address the underlying mechanisms.

The rise in popularity of low carbohydrate diets, especially in the United States and other Western countries is based on studies which have reported higher levels of insulin and triglyceride and lower levels of HDL cholesterol for high carbohydrate diets when compared with lower carbohydrate, higher fat diets (Hu et al., 2012, Kodama et al., 2009., Mensink et al., 2003). The profile of cardiometabolic risk factors measured on the three diets in the present study suggests that the traditional high carbohydrate diet characteristic of the dietary patterns in China is associated with an overall lower risk of cardiovascular diseases than the higher fat diets which are relatively high in unsaturated vegetable derived fatty acids (soybean oil). As expected, replacement of fat with carbohydrate was associated with lowered HDL cholesterol levels, but no significant differences in ratio of total and HDL cholesterol. Diet induced lowering of HDL cholesterol results in more rapid clearance of HDL and decreased transport of HDL apolipoproteins, which may not confer the same risk of atherosclerosis as do low HDL cholesterol levels in subjects consuming a high-fat diet (Connor and Connor, 1997). Several studies confirm that eating a low-fat diet is not associated with an increased incidence of CHD despite a reduction in HDL (Connor et al., 1978, Knuiman et al., 1982, Ornish et al., 1998). The relatively high baseline HDL concentration (1.48 mmol/L), provides some additional reassurance that this may not be an important determinant of cardiovascular risk in the population we have studied. Furthermore, the fact that glucose and insulin levels were not significantly different for the high and lower carbohydrate diets and that body weight reduced with increasing carbohydrate intake suggests that among the Chinese, traditional high carbohydrate diets are unlikely to be associated with an increased risk of diabetes.

Our trial has several limitations. We relied on self-reported dietary intake in the intervention period. However, all food was provided throughout the 6 month intervention period and one meal each day was consumed under supervision. All of these measures ensured a high likelihood of achieving dietary targets. We also note that this limitation would apply to all dietary intervention trials in which participants were not confined in a metabolic facility. In addition, the fact that lipid measurements altered in the expected direction with increasing intakes of carbohydrate might be considered as providing indirect evidence of compliance with the test diets. One might argue that compared with changes seen in weight-loss trials among obese subjects, the relatively small benefit observed in terms of body weight and other measurements of interest may be of little clinical relevance. However, the effects are potentially considerable among the non-obese population, especially when translated into population health outcomes in a country that is in a state of nutrition transition. Only non-obese healthy subjects were included, but based on current trends in China many of them are likely to become obese and develop cardiometabolic disease in the future. Therefore, we would suggest that our results are of considerable potential relevance.

The strengths of the present study include the study design, the relatively long duration of the intervention, the similar results observed when analyzing the data according to intention-to-treat and per-protocol or whether including weight change or not, and high retention rates, all of which enhance our confidence in the findings.

In conclusion, among non-obese healthy Chinese, a low fat, relatively high carbohydrate diet, similar in macronutrient composition to traditional dietary patterns in China appears to be less likely to promote excessive weight gain and be associated with a lower cardiometabolic risk profile than a diet more typical of that eaten in Western countries. Our findings in conjunction with all other available evidence suggest that the Chinese population should be discouraged from following the continuing trend towards increasing dietary fat intake at the expense of carbohydrates.

## Funding Source

This study was funded by the National Basic Research Program of China (2015CB553604). The funder of the study had no role in study design, data collection, data analysis, data interpretation, or writing of the manuscript. The corresponding author had full access to all data and had final responsibility for the decision to submit for publication.

## Conflicts of Interests

The authors declare no conflicts of interest.

## Author Contributions

D.L. obtained the study funding. D.L., Y.W. and F.W. conceived and designed the study. Y.W. and F.W. developed the protocol and conducted the study with all other authors. D.J., J.L., J.Z., and J.G. enrolled the participants. D.L. and J.Y. provided administrative support. Y.W., F.W., Z.J., T.H., and J.Z. analyzed the data. Y.W. and F.W. wrote the manuscript. D.L. and J.M. revised the manuscript. All authors interpreted the data and revised the manuscript for important intellectual content and approved the final draft.

## Appendix A Supplementary Data

Supplementary tables and figures Image 1

## References

[bb0005] Adair L.S., Gordon-Larsen P., Du S.F., Zhang B., Popkin B.M. (2014). The emergence of cardiometabolic disease risk in Chinese children and adults: consequences of changes in diet, physical activity and obesity. Obes. Rev..

[bb0010] Austin G.L., Ogden L.G., Hill J.O. (2011). Trends in carbohydrate, fat, and protein intakes and association with energy intake in normal-weight, overweight, and obese individuals: 1971–2006. Am. J. Clin. Nutr..

[bb0015] Connor W.E., Connor S.L. (1997). Should a low-fat, high-carbohydrate diet be recommended for everyone? The case for a low-fat, high-carbohydrate diet. N. Engl. J. Med..

[bb0020] Connor W.E., Cerqueira M.T., Connor R.W., Wallace R.B., Malinow M.R., Casdorph H.R. (1978). The plasma lipids, lipoproteins, and diet of the Tarahumara indians of Mexico. Am. J. Clin. Nutr..

[bb0025] Chinese Nutrition Society (2014). Chinese Dietary Reference Intakes.

[bb0030] Campbell T.M. (2004). The China Study: The most Comprehensive Study of Nutrition Ever Conducted and the Startling Implications for Diet, Weight Loss and Long-Term Health.

[bb0035] Du S.F., Wang H.J., Zhang B., Zhai F.Y., Popkin B.M. (2014). China in the period of transition from scarcity and extensive undernutrition to emerging nutrition-related non-communicable diseases, 1949–1992. Obes. Rev..

[bb0040] Gardner C.D., Kiazand A., Alhassan S., Kim S., Stafford R.S., Balise R.R., Kraemer H.C., King A.C. (2007). Comparison of the Atkins, Zone, Ornish, and LEARN diets for change in weight and related risk factors among overweight premenopausal women: the A TO Z weight loss study: a randomized trial. JAMA.

[bb0045] Hu F.B. (2010). Are refined carbohydrates worse than saturated fat?. Am. J. Clin. Nutr..

[bb0050] Hooper L., Abdelhamid A., Bunn D., Brown T., Summerbell C.D., Skeaff C.M. (2015). Effects of total fat intake on body weight. Cochrane Database Syst. Rev..

[bb0055] Hu T., Mills K.T., Yao L., Demanelis K., Eloustaz M., Yancy W.J., Kelly T.N., He J., Bazzano L.A. (2012). Effects of low-carbohydrate diets versus low-fat diets on metabolic risk factors: a meta-analysis of randomized controlled clinical trials. Am. J. Epidemiol..

[bb0060] Hariri N., Thibault L. (2010). High-fat diet-induced obesity in animal models. Nutr. Res. Rev..

[bb0065] Halton T.L., Willett W.C., Liu S., Manson J.E., Albert C.M., Rexrode K., Hu F.B. (2006). Low-carbohydrate-diet score and the risk of coronary heart disease in women. N. Engl. J. Med..

[bb0070] How Can I Minimize Loss of Data due to Missing Observations in a Repeated Measures ANOVA? UCLA: Statistical Consulting Group. Available from: http://stats.idre.ucla.edu/sas/faq/how-can-i-minimize-loss-of-data-due-to-missing-observations-in-a-repeated-measures-anova/ (Accessed November 22, 2016).

[bb0075] Jenkins D.J., Wong J.M., Kendall C.W., Esfahani A., Ng V.W., Leong T.C., Faulkner D.A., Vidgen E., Greaves K.A., Paul G., Singer W. (2009). The effect of a plant-based low-carbohydrate ("Eco-Atkins") diet on body weight and blood lipid concentrations in hyperlipidemic subjects. Arch. Intern. Med..

[bb0080] Kodama S., Saito K., Tanaka S., Maki M., Yachi Y., Sato M., Sugawara A., Totsuka K., Shimano H., Ohashi Y., Yamada N., Sone H. (2009). Influence of fat and carbohydrate proportions on the metabolic profile in patients with type 2 diabetes: a meta-analysis. Diabetes Care.

[bb0085] Knuiman J.T., West C.E., Burema J. (1982). Serum total and high density lipoprotein cholesterol concentrations and body mass index in adult men from 13 countries. Am. J. Epidemiol..

[bb0090] Livesey G., Taylor R., Livesey H., Liu S. (2013). Is there a dose-response relation of dietary glycemic load to risk of type 2 diabetes? Meta-analysis of prospective cohort studies. Am. J. Clin. Nutr..

[bb0095] Macdiarmid J., Blundell J. (1998). Assessing dietary intake: who, what and why of under-reporting. Nutr. Res. Rev..

[bb0100] Mozaffarian D., Hao T., Rimm E.B., Willett W.C., Hu F.B. (2011). Changes in diet and lifestyle and long-term weight gain in women and men. N. Engl. J. Med..

[bb0105] Mensink R.P., Zock P.L., Kester A.D., Katan M.B. (2003). Effects of dietary fatty acids and carbohydrates on the ratio of serum total to HDL cholesterol and on serum lipids and apolipoproteins: a meta-analysis of 60 controlled trials. Am. J. Clin. Nutr..

[bb0110] Ministry of Health: Bureau of Disease Control and Prevention (2011). China NCD Report 2011 Beijing, China.

[bb0115] Ornish D., Scherwitz L.W., Billings J.H., Brown S.E., Gould K.L., Merritt T.A., Sparler S., Armstrong W.T., Ports T.A., Kirkeeide R.L., Hogeboom C., Brand R.J. (1998). Intensive lifestyle changes for reversal of coronary heart disease. JAMA.

[bb0120] Popkin B.M., Paeratakul S., Zhai F., Ge K. (1995). Dietary and environmental correlates of obesity in a population study in China. Obes. Res..

[bb0125] Turnbaugh P.J., Ley R.E., Mahowald M.A., Magrini V., Mardis E.R., Gordon J.I. (2006). An obesity-associated gut microbiome with increased capacity for energy harvest. Nature.

[bb0130] Te Morenga L., Mallard S., Mann J. (2013). Dietary sugars and body weight: systematic review and meta-analyses of randomized controlled trials and cohort studies. BMJ.

[bb0135] Villegas R., Liu S., Gao Y.T., Yang G., Li H., Zheng W., Shu X.O. (2007). Prospective study of dietary carbohydrates, glycemic index, glycemic load, and incidence of type 2 diabetes mellitus in middle-aged Chinese women. Arch. Intern. Med..

[bb9000] Wan Y., Wang F.L., Yuan J.H., Li D. (2017). Optimal dietary macronutrient distribution in China (ODMDC): a randomised controlled-feeding trial protocol. Asia Pac. J. Clin. Nutr..

[bb0140] World Health Organization: Department of Chronic Diseases and Health Promotion Global Physical Activity Questionnaire (GPAQ) Analysis Guide. http://www.who.int/chp/steps/resources/GPAQ_Analysis_Guide.pdf.

[bb0145] Yu D., Shu X.O., Li H., Xiang Y.B., Yang G., Gao Y.T., Zheng W., Zhang X. (2013). Dietary carbohydrates, refined grains, glycemic load, and risk of coronary heart disease in Chinese adults. Am. J. Epidemiol..

[bb0150] Zhai F.Y., Du S.F., Wang Z.H., Zhang J.G., Du W.W., Popkin B.M. (2014). Dynamics of the Chinese diet and the role of urbanicity, 1991–2011. Obes. Rev..

