# Supplementary material for: Effects of Macronutrient Distribution on Weight and Related Cardiometabolic Profile in Healthy Non-Obese Chinese: A 6-month, Randomized Controlled-Feeding Trial
Source: eBioMedicine. 2017 Jun 20;22:200–7. doi: 10.1016/j.ebiom.2017.06.017 (PMC5672080; doi:10.1016/j.ebiom.2017.06.017)
Supplement: Supplementary file 1 — Supplementary tables and figures [file mmc1.docx]

**Supplementary Appendix**

**Table S1. Protocol for the screening, run-in periods, and randomization**

**Table S2. Primary outcome**

**Table S3. Secondary outcomes**

**Fig. S1. Differences in outcomes according to whether multiple imputation or mixed-model or the observed value was used at 6 months**

**Fig. S2. Participant satisfaction and percent of perfect adherence**

**Fig. S3. 3-day dietary intake and physical activity at each time point**

**Table S1. Protocol for the screening, run-in periods, and randomization**

| Screening visit 1 by telephone or internet interview |
| --- |
| Informed consent for screening and run-in periods |
| Major eligibility questions |
| Medical history questionnaire |
| General dietary questionnaire |
| Screening visit 2 by face to face interview* |
| 3-day food records |
| Physical activity questionnaire  Breakfast accessibility test |
| Screening visit 3† |
| Weight, height, waist circumferences, hip circumferences, blood pressure |
| 10-hour fasting blood sample for eligibility testing |
| Run-in period (7 days) |
| Feed the subjects the moderate fat, moderate carbohydrate diet |
| Lunch and dinner eaten on a dinning site |
| Prepared breakfast distributed for consumption off-site |
| Daily diet diaries |
| Compliance assessment, and adherence counseling as needed‡ |
| Side effects questionnaires |
| Randomization takes place at the beginning of day 8 |

*The screening visit 2 was 7 days after previous visit. All potential participants were informed to send the 3-day food records and physical activity questionnaire to our working email when finished. †The screening visit 3 was 10 days after previous visit. ‡Adherence to the dietary intervention is assessed by observation, self-report of study subjects. During the provision of on-site meals, adherence is assessed by direct observation of the number of missed meals, the quantity of the uneaten food and the subjective judgment of the clinic staff. Self-report measures include daily diary that records any deviations throughout the trial in addition to satisfaction level.

**Table S2. Primary outcome**

| **Primary outcome** | **Change from baseline (95% CI)** | | | | | |
| --- | --- | --- | --- | --- | --- | --- |
|  | **Month 1** | **Month 2** | **Month 3** | **Month 4** | **Month 5** | **Month 6** |
| ITT*  Weight change (kg) |  |  |  |  |  |  |
| LF-HC | -1.5 (-1.7 to -1.3) | -2.0 (-2.1 to -1.8) | -1.8 (-2.0 to -1.7) | -1.8 (-2.0 to -1.6) | -1.8 (-2.0 to -1.6) | -1.6 (-1.8 to -1.4) |
| MF-MC | -1.0 (-1.2 to -0.8) | -1.4 (-1.6 to -1.2) | -1.5 (-1.7 to -1.3) | -1.3 (-1.5 to -1.1) | -1.2 (-1.4 to -1.0) | -1.1 (-1.3 to -0.9) |
| HF-LC | -0.9 (-1.1 to -0.7) | -1.1 (-1.3 to -0.9) | -1.2 (-1.4 to -1.1) | -1.0 (-1.2 to -0.8) | -1.0 (-1.2 to -0.8) | -0.9 (-1.1 to -0.6) |
| PP  Weight change (kg) |  |  |  |  |  |  |
| LF-HC | -1.5 (-1.7 to -1.4) | -1.9 (-2.1 to -1.7) | -1.8 (-2.0 to -1.6) | -1.7 (-1.9 to -1.5) | -1.7 (-1.9 to -1.5) | -1.6 (-1.7 to -1.4) |
| MF-MC | -1.0 (-1.2 to -0.8) | -1.3 (-1.6 to -1.1) | -1.5 (-1.7 to -1.2) | -1.3 (-1.6 to -1.0) | -1.2 (-1.5 to -0.9) | -1.1 (-1.3 to -0.8) |
| HF-LC | -1.0 (-1.3 to -0.8) | -1.3 (-1.5 to -1.0) | -1.5 (-1.7 to -1.2) | -1.1 (-1.4 to -0.9) | -1.2 (-1.5 to -0.9) | -1.0 (-1.3 to -0.7) |

ITT=intention-to-treat. LF-HC=lower fat, higher carbohydrate. MF-MC= moderate fat, moderate carbohydrate. HF-LC=higher fat, lower carbohydrate. PP=per-protocol. *Data are based on mixed-model analysis of variance

**Table S3. Secondary outcomes***

| **Secondary outcomes** | **Change from baseline (95% CI)** | | | | | |
| --- | --- | --- | --- | --- | --- | --- |
|  | **Month 1** | **Month 2** | **Month 3** | **Month 4** | **Month 5** | **Month 6** |
| Waist circumference (cm) |  |  |  |  |  |  |
| LF-HC | -0.8 (-1.0 to -0.7) | -1.1 (-1.3 to -1.0) | -1.1 (-1.3 to -1.0) | -1.2 (-1.4 to -1.0) | -1.1 (-1.3 to -1.0) | -1.1 (-1.3 to -0.9) |
| MF-MC | -0.4 (-0.6 to -0.3) | -0.6 (-0.7 to -0.4) | -0.7 (-0.8 to -0.5) | -0.6 (-0.8 to -0.5) | -0.6 (-0.7 to -0.5) | -0.6 (-0.7 to -0.4) |
| HF-LC | -0.3 (-0.5 to -0.2) | -0.5 (-0.6 to -0.3) | -0.5 (-0.6 to -0.4) | -0.5 (-0.6 to -0.4) | -0.4 (-0.5 to -0.3) | -0.3 (-0.5 to -0.2) |
| Total cholesterol (mmol/L) |  |  |  |  |  |  |
| LF-HC | -0.31 (-0.39 to -0.23) | -0.35 (-0.43 to -0.27) | -0.33 (-0.41 to -0.25) | -0.28 (-0.36 to -0.19) | -0.26 (-0.34 to -0.18) | -0.20 (-0.28 to -0.12) |
| MF-MC | -0.19 (-0.27 to -0.11) | -0.21 (-0.29 to -0.13) | -0.11 (-0.19 to -0.03) | -0.11 (-0.19 to -0.03) | -0.05 (-0.14 to 0.03) | -0.02 (-0.10 to 0.06) |
| HF-LC | -0.14 (-0.22 to -0.07) | -0.17 (-0.25 to -0.10) | -0.17 (-0.25 to -0.09) | -0.03 (-0.11 to 0.05) | -0.02 (-0.10 to 0.07) | 0.06 (-0.03 to 0.14) |
| LDL cholesterol (mmol/L) |  |  |  |  |  |  |
| LF-HC | -0.2 5(-0.3 1to -0.19) | -0.29 (-0.35 to -0.23) | -0.29 (-0.35 to -0.22) | -0.24 (-0.3 to -0.18) | -0.22 (-0.29 to -0.16) | -0.17 (-0.24 to -0.11) |
| MF-MC | -0.14 (-0.2 to -0.08) | -0.16 (-0.22 to -0.1) | -0.18 (-0.24 to -0.11) | -0.1 4(-0.21 to -0.08) | -0.09 (-0.15 to -0.02) | -0.07 (-0.14 to -0.01) |
| HF-LC | -0.1 (-0.16 to -0.04) | -0.16 (-0.22 to -0.1) | -0.19 (-0.25 to -0.13) | -0.09 (-0.15 to -0.02) | -0.05 (-0.11 to 0.01) | -0.01 (-0.07 to 0.06) |
| HDL cholesterol (mmol/L) |  |  |  |  |  |  |
| LF-HC | -0.08 (-0.11 to -0.04) | -0.11 (-0.14 to -0.07) | -0.11 (-0.14 to -0.08) | -0.08 (-0.11 to -0.05) | -0.08 (-0.12 to -0.05) | -0.07 (-0.11 to -0.04) |
| MF-MC | -0.06 (-0.09 to -0.03) | -0.08 (-0.11 to -0.05) | -0.06 (-0.10 to -0.03) | -0.05 (-0.09 to -0.02) | -0.02 (-0.06 to 0.01) | 0.00 (-0.03 to 0.04) |
| HF-LC | -0.03 (-0.06 to 0.00) | -0.07 (-0.11 to -0.04) | -0.07 (-0.10 to -0.03) | -0.04 (-0.0 7 to 0.00) | -0.01 (-0.05 to 0.02) | 0.03 (-0.01 to 0.06) |
| Non-HDL cholesterol (mmol/L) |  |  |  |  |  |  |
| LF-HC | -0.24 (-0.31 to -0.17) | -0.24 (-0.31 to -0.17) | -0.22 (-0.29 to -0.15) | -0.19 (-0.26 to -0.12) | -0.18 (-0.25 to -0.11) | -0.13 (-0.20 to -0.06) |
| MF-MC | -0.13 (-0.2 to -0.07) | -0.13 (-0.2 to -0.06) | -0.04 (-0.11 to 0.03) | -0.06 (-0.13 to 0.01) | -0.03 (-0.10 to 0.04) | -0.02 (-0.09 to 0.05) |
| HF-LC | -0.12 (-0.18 to -0.05) | -0.1 (-0.17 to -0.03) | -0.1 (-0.17 to -0.03) | 0.01 (-0.06 to 0.08) | 0.00 (-0.07 to 0.07) | 0.03 (-0.04 to 0.11) |
| Total/HDL cholesterol |  |  |  |  |  |  |
| LF-HC | -0.06 (-0.12 to 0.00) | -0.04 (-0.11 to 0.02) | -0.00 (-0.07 to 0.07) | -0.02 (-0.09 to 0.04) | -0.03 (-0.09 to 0.04) | 0.04 (-0.02 to 0.11) |
| MF-MC | 0.00 (-0.07 to 0.06) | 0.03 (-0.03 to 0.10) | 0.08 (0.01 to 0.15) | 0.06 (-0.01 to 0.12) | 0.00 (-0.07 to 0.07) | 0.02 (-0.05 to 0.09) |
| HF-LC | -0.04 (-0.10 to 0.02) | 0.03 (-0.03 to 0.10) | 0.00 (-0.06 to 0.07) | 0.03 (-0.04 to 0.10) | 0.00 (-0.07 to 0.07) | -0.01 (-0.08 to 0.06) |
| Triglycerides (mmol/L) |  |  |  |  |  |  |
| LF-HC | 0.02 (-0.03 to 0.06) | 0.08 (0.04 to 0.13) | 0.21 (0.16 to 0.25) | 0.16 (0.12 to 0.21) | 0.14 (0.09 to 0.18) | 0.13 (0.08 to 0.18) |
| MF-MC | -0.01 (-0.06 to 0.03) | 0.07 (0.03 to 0.12) | 0.21 (0.16 to 0.25) | 0.13 (0.08 to 0.18) | 0.11 (0.06 to 0.16) | 0.07 (0.02 to 0.12) |
| HF-LC | -0.03 (-0.07 to 0.02) | 0.04 (-0.01 to 0.09) | 0.15 (0.1 to 0.19) | 0.10 (0.05 to 0.14) | 0.07 (0.02 to 0.11) | 0.06 (0.02 to 0.11) |
| Apolipoprotein A1 (mmol/L) |  |  |  |  |  |  |
| LF-HC | -0.08 (-0.11 to -0.06) | -0.11 (-0.14 to -0.08) | -0.11 (-0.14 to -0.08) | -0.08 (-0.11 to -0.05) | -0.07 (-0.1 to -0.05) | -0.06 (-0.08 to -0.03) |
| MF-MC | -0.06 (-0.09 to -0.04) | -0.10 (-0.12 to -0.07) | -0.07 (-0.09 to -0.04) | -0.06 (-0.08 to -0.03) | -0.05 (-0.08 to -0.02) | -0.03 (-0.06 to 0.00) |
| HF-LC | -0.03 (-0.06 to -0.01) | -0.07 (-0.1 to -0.05) | -0.08 (-0.10 to -0.05) | -0.04 (-0.06 to -0.01) | -0.02 (-0.05 to 0.01) | 0.00 (-0.03 to 0.03) |
| Apolipoprotein B (mmol/L) |  |  |  |  |  |  |
| LF-HC | -0.06 (-0.08 to -0.04) | -0.06 (-0.08 to -0.05) | -0.07 (-0.09 to -0.06) | -0.06 (-0.08 to -0.05) | -0.05 (-0.06 to -0.03) | -0.03 (-0.05 to -0.02) |
| MF-MC | -0.03 (-0.05 to -0.01) | -0.04 (-0.05 to -0.02) | -0.04 (-0.06 to -0.03) | -0.03 (-0.05 to -0.01) | -0.02 (-0.04 to -0.01) | -0.01 (-0.03 to 0.01) |
| HF-LC | -0.02 (-0.04 to 0.00) | -0.03 (-0.05 to -0.02) | -0.03 (-0.05 to -0.01) | -0.02 (-0.04 to 0.00) | -0.01 (-0.03 to 0.01) | 0.00 (-0.01 to 0.02) |
| SBP (mm Hg) |  |  |  |  |  |  |
| LF-HC | -2.9 (-3.9 to -1.9) | -2.7 (-3.7 to -1.7) | -2.3 (-3.3 to -1.2) | -2.7 (-3.6 to -1.8) | -2.8 (-3.7 to -1.9) | -2.6 (-3.6 to -1.6) |
| MF-MC | -1.9 (-2.9 to -0.9) | -1.7 (-2.7 to -0.7) | -1.4 (-2.4 to -0.3) | -1.2 (-2.3 to -0.2) | -1.9 (-2.9 to -0.8) | -2.1 (-3.1 to -1.0) |
| HF-LC | -1.8 (-2.8 to -0.8) | -2.1 (-3.0 to -1.1) | -1.3 (-2.4 to -0.3) | -2.0 (-3.1 to -1.1) | -1.8 (-2.9 to -0.7) | -2.4 (-3.5 to -1.3) |
| DBP (mm Hg) |  |  |  |  |  |  |
| LF-HC | -1.1 (-1.6 to -0.5) | -1.3 (-1.8 to -0.7) | -1.2 (-1.8 to -0.6) | -1.4 (-1.9 to -0.8) | -1.3 (-1.8 to -0.7) | -1.3 (-1.9 to -0.7) |
| MF-MC | -1.2 (-1.7 to -0.6) | -0.6 (-1.2 to 0.1) | -0.5 (-1.1 to 0.0) | -0.9 (-1.4 to -0.3) | -1.1 (-1.7 to -0.5) | -1.0 (-1.7 to -0.5) |
| HF-LC | -1.1 (-1.7 to -0.5) | -1.1 (-1.7 to -0.6) | -0.6 (-1.1 to 0.0) | -0.8 (-1.3 to -0.2) | -1.1 (-1.6 to -0.5) | -1.1 (-1.7 to -0.5) |
| Glucose (mmol/L) |  |  |  |  |  |  |
| LF-HC | -0.02 (-0.08 to 0.04) | 0.01 (-0.05 to 0.07) | 0.00 (-0.06 to 0.05) | 0.00 (-0.06 to 0.06) | 0.02 (-0.04 to 0.08) | 0.03 (-0.03 to 0.09) |
| MF-MC | -0.03 (-0.09 to 0.02) | -0.02 (-0.08 to 0.03) | 0.00 (-0.06 to 0.06) | 0.00 (-0.06 to 0.06) | 0.00 (-0.06 to 0.06) | 0.00 (-0.06 to 0.07) |
| HF-LC | -0.01 (-0.07 to 0.04) | -0.04 (-0.1 to 0.01) | -0.05 (-0.11 to 0.01) | 0.00 (-0.06 to 0.06) | -0.02 (-0.08 to 0.04) | -0.01 (-0.08 to 0.05) |
| Insulin (pmol/L) |  |  |  |  |  |  |
| LF-HC | -5.93 (-10.47 to -1.39) | -4.00 (-8.60 to 0.60) | -3.55 (-8.20 to 1.10) | -3.68 (-8.36 to 1.00) | -3.83 (-8.56 to 0.90) | -2.53 (-7.31 to 2.25) |
| MF-MC | -4.10 (-8.60 to 0.41) | -3.23 (-7.81 to 1.35) | -2.70 (-7.33 to 1.92) | -3.14 (-7.84 to 1.55) | -3.81 (-8.54 to 0.92) | -3.35 (-8.15 to 1.44) |
| HF-LC | -1.05 (-5.57 to 3.47) | -0.41 (-5.01 to 4.19) | -2.01 (-6.69 to 2.68) | -1.49 (-6.22 to 3.24) | -1.91 (-6.73 to 2.90) | -2.82 (-7.75 to 2.11) |
| GSP (μmol/L) |  |  |  |  |  |  |
| LF-HC | -5.7 (-7.4 to -3.9) | -5.2 (-7.0 to -3.4) | -4.1 (-5.9 to -2.3) | -3.5 (-5.3 to -1.7) | -4.3 (-6.1 to -2.5) | -3.5 (-5.3 to -1.6) |
| MF-MC | -5.1 (-6.8 to -3.4) | -3.7 (-5.4 to -1.9) | -3.0 (-4.9 to -1.4) | -4.2 (-6.0 to -2.4) | -4.0 (-5.9 to -2.2) | -1.6 (-3.4 to 0.3) |
| HF-LC | -4.6 (-6.3 to -2.8) | -4.7 (-6.4 to -2.9) | -3.4 (-5.2 to -1.6) | -3.6 (-5.4 to -1.8) | -4.4 (-6.3 to -2.6) | -3.2 (-5.1 to -1.3) |
| Adiponectin (mg/L) |  |  |  |  |  |  |
| LF-HC | 0.2 (-0.2 to 0.7) | 0.2 (-0.3 to 0.6) | 0.3 (-0.1 to 0.8) | 0.1 (-0.4 to 0.5) | 0.3 (-0.2 to 0.8) | 0.3 (-0.1 to 0.8) |
| MF-MC | 0.2 (-0.2 to 0.7) | 0.1 (-0.4 to 0.5) | 0.1 (-0.4 to 0.5) | 0.4 (-0.1 to 0.9) | 0.1 (-0.4 to 0.5) | 0.3 (-0.1 to 0.8) |
| HF-LC | -0.1 (-0.6 to 0.3) | -0.2 (-0.7 to 0.2) | 0.1 (-0.3 to 0.6) | 0.2 (-0.2 to 0.7) | 0.0 (-0.5 to 0.4) | 0.2 (-0.3 to 0.6) |
| Leptin (μg/L) |  |  |  |  |  |  |
| LF-HC | -0.5 (-0.9 to -0.1) | -0.6 (-1.0 to -0.2) | -0.4 (-0.8 to 0.0) | -0.4 (-0.8 to 0.0) | -0.5 (-0.9 to 0.0) | -0.4 (-0.8 to 0.0) |
| MF-MC | -0.4 (-0.7 to 0.1) | -0.3 (-0.7 to 0.1) | -0.5 (-0.9 to -0.1) | -0.3 (-0.7 to 0.0) | -0.4 (-0.8 to 0.0) | -0.4 (-0.8 to 0.0) |
| HF-LC | 0.0 (-0.4 to 0.4) | 0.0 (-0.4 to 0.4) | 0.0 (-0.4 to 0.4) | -0.2 (-0.6 to 0.2) | -0.1 (-0.5 to 0.3) | -0.3 (-0.7 to 0.1) |

LF-HC=lower fat, higher carbohydrate. MF-MC= moderate fat, moderate carbohydrate. HF-LC=higher fat, lower carbohydrate. LDL=low-density lipoprotein. HDL=high-density lipoprotein. SBP=systolic blood pressure. DBP=diastolic blood pressure. GSP=glycated serum protein. *Data are based on mixed-model analysis of variance

**
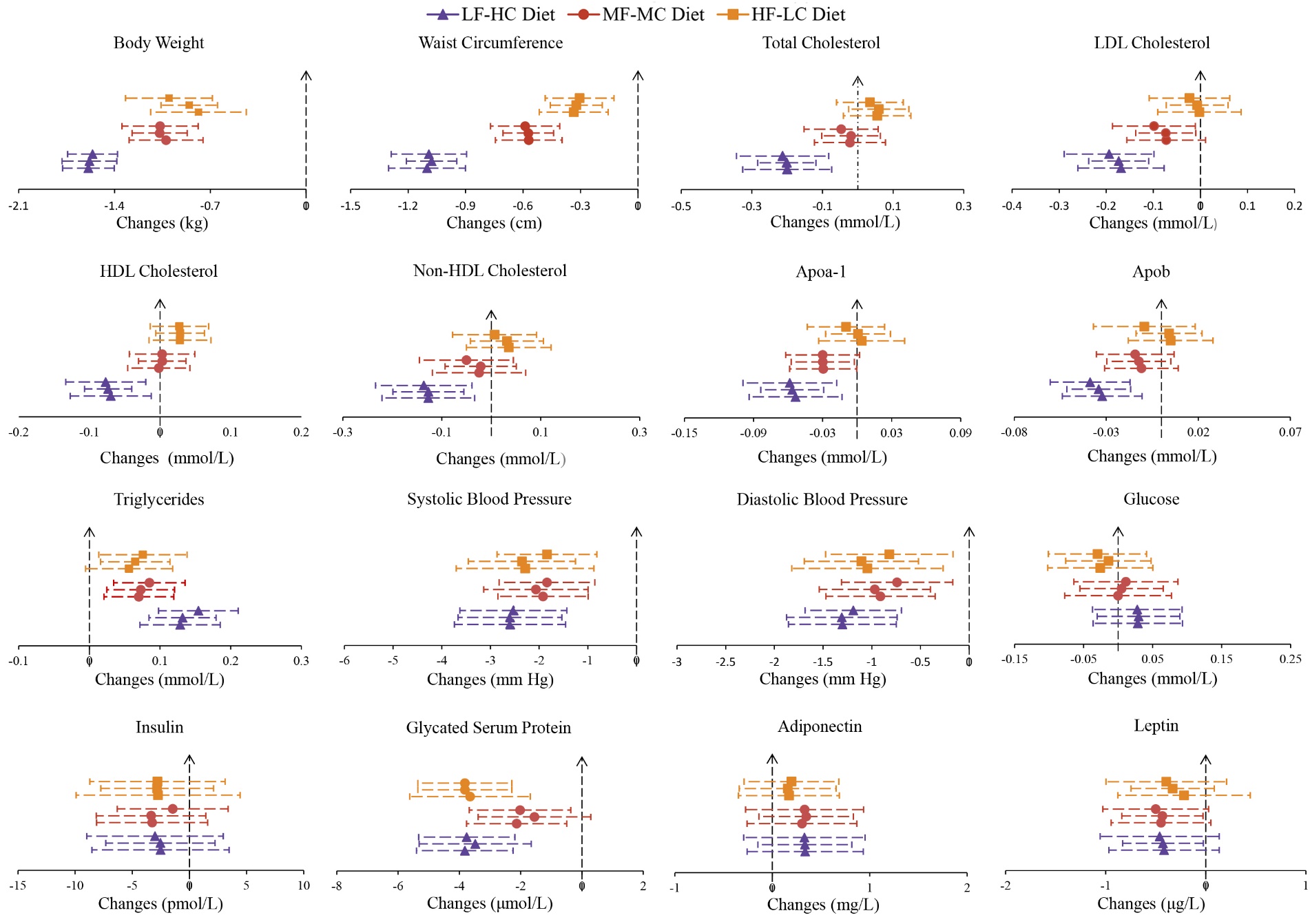
Fig. S1. Differences in outcomes according to whether multiple imputation or mixed-model or the observed value was used at 6 months**

LF-HC=lower fat, higher carbohydrate. MF-MC= moderate fat, moderate carbohydrate. HF-LC=higher fat, lower carbohydrate. Multiple imputation analyses the effect on the results of the failure of some of the participants to complete the study diets. We conducted sensitivity analysis for all outcomes according to whether multiple imputation, mixed-model or the observed value (complete case) was used at 6 months. There was no qualitative difference of multiple imputation compared with values from mixed-model and complete case analysis. Three lines in each diet group denote multiple imputation estimate, mixed-model estimate, and complete case estimate, from top to bottom respectively. Multiple imputation was performed using “proc mi” and “proc mianalyze” in SAS 9.3, with five imputations to impute missing responses.


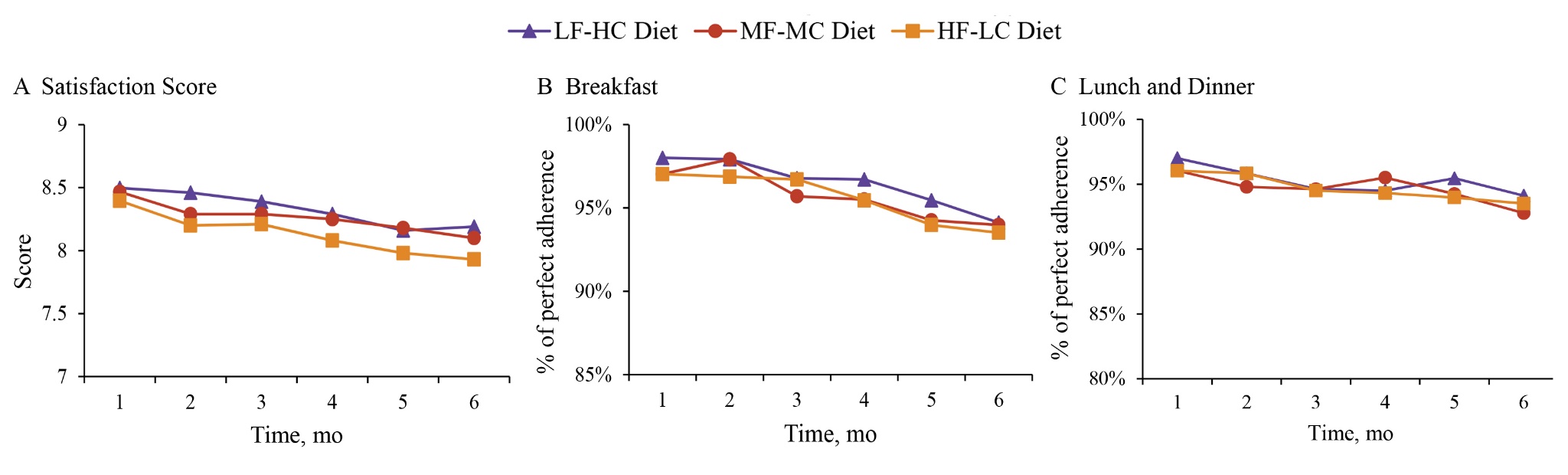
**Fig. S2. Participant satisfaction and percent of perfect adherence**

LF-HC=lower fat, higher carbohydrate. MF-MC= moderate fat, moderate carbohydrate. HF-LC=higher fat, lower carbohydrate.


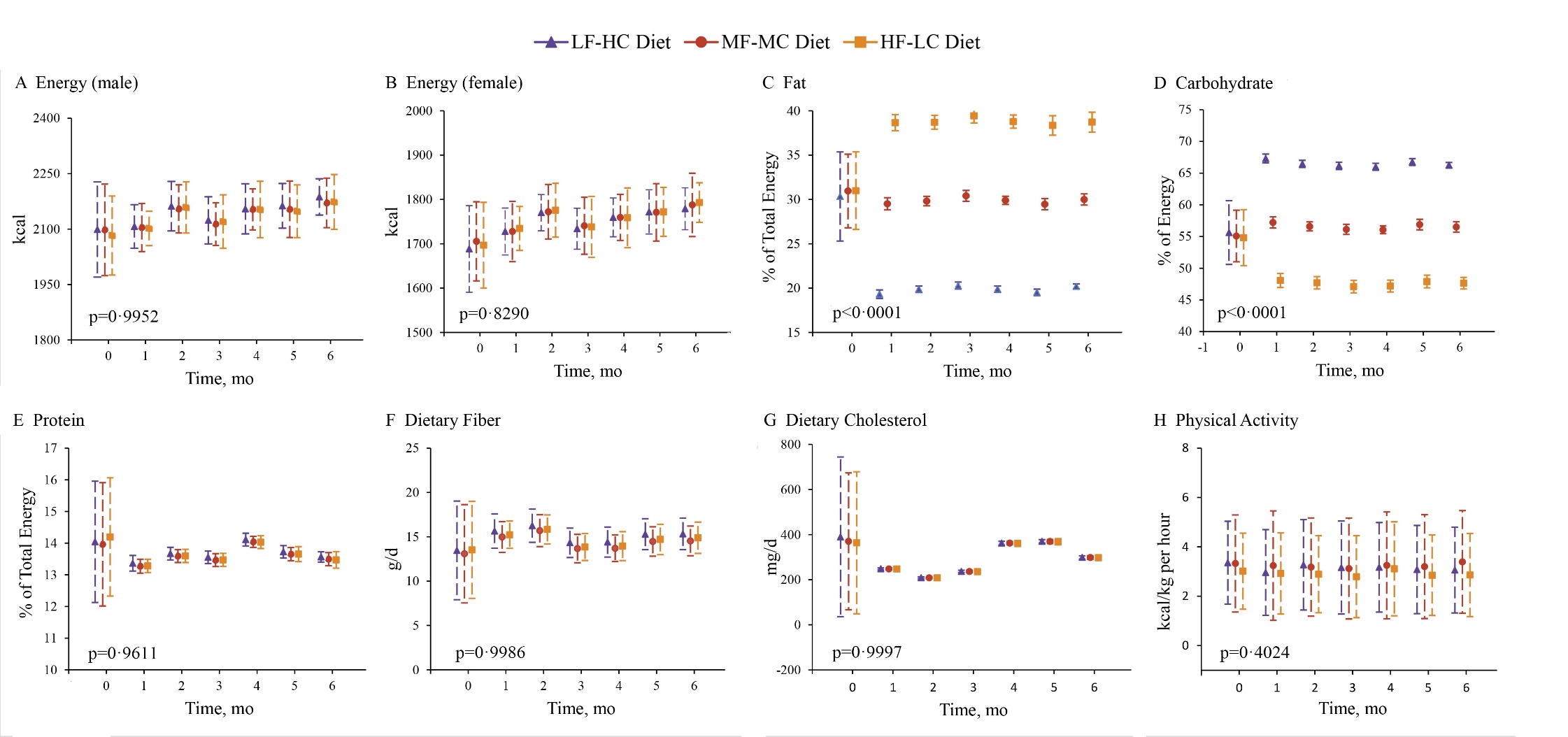
**Fig. S3. 3-day dietary intake and physical activity at each time point***

LF-HC=lower fat, higher carbohydrate. MF-MC= moderate fat, moderate carbohydrate. HF-LC=higher fat, lower carbohydrate. *Panel A and B shows the mean energy intake in male and female subjects, respectively. Panel C for the fat intake (% of total energy), Panel D for the carbohydrate intake (% of total energy), Panel E for the protein intake (% of total energy), Panel F for the dietary fiber intake, Panel G for the dietary cholesterol intake, Panel H for the physical activity. The dietary intake and physical activity levels over time were evaluated by mixed-model analysis of variance. Age, sex, and study center were included as covariates in all analyses. The p value at the lower left of each panel indicates the group × time interaction with no imputation of missing data. From Panel A to Panel G, data were included from 307 participants at baseline, 296 participants at 1 month, 282 participants at 2 months, 271 participants at 3 months, 262 participants at 4 months, 253 participants at 5 months and 240 participants at 6 months. In Panel H, available data from baseline to 6 months were 307, 302, 286, 276, 268, 257 and 244, respectively.
